# Determining transition readiness in Swiss childhood cancer survivors – a feasibility study

**DOI:** 10.1186/s12885-021-07787-8

**Published:** 2021-01-21

**Authors:** Maria Otth, Patrick Wechsler, Sibylle Denzler, Henrik Koehler, Katrin Scheinemann

**Affiliations:** 1 Division of Oncology-Hematology, Department of Pediatrics, Kantonsspital Aarau, Tellstrasse 25, 5001 Aarau, Switzerland; 2 Institute of Social and Preventive Medicine, University of Bern, Bern, Switzerland; 3 University of Basel, Basel, Switzerland; 4 Department of Pediatrics, Kantonsspital Aarau, Aarau, Switzerland; 5 Department of Pediatrics, McMaster Children’s Hospital and McMaster University, Hamilton, Canada

**Keywords:** Transition, Childhood cancer survivors, Readiness, Scales

## Abstract

**Background:**

The successful transition of childhood cancer survivors (CCSs) from pediatric to adult long-term follow-up care is a critical phase, and determining the right time point can be challenging. We assessed the feasibility of the use of existing transition readiness tools in the context of the Swiss health care system, assessed partly transition readiness in Swiss CCSs, and compared our findings with Canadian CCSs for which these tools were originally developed.

**Methods:**

We officially translated the Cancer Worry Scale (CWS) and Self-Management Skill Scale (SMSS) into German and integrated them into this cross-sectional study. We included CCSs attending the long-term follow-up (LTFU) clinic in the Division of Oncology-Hematology, Department of Pediatrics, Kantonsspital Aarau. We used descriptive statistics to describe transition readiness.

**Results:**

We randomly recruited 50 CCSs aged ≥18 years at participation. The CCSs had a median CWS score of 62 (interquartile range 55–71), indicating a moderate level of cancer-related worry. Despite high self-management skills, some answers showed a dependency of CCSs on their parents. Our experience shows that the CWS and SMSS are easy for Swiss CCSs to use, understand, and complete. The interpretation of the results must take differences in health care systems between countries into account.

**Conclusions:**

The translated CWS and SMSS are appropriate additional measures to assess transition readiness in CCSs. These scales can be used longitudinally to find the individual time point for transition and the completion by CCSs enables the health care team to individualize the transition process and to support the CCSs according to their individual needs.

**Supplementary Information:**

The online version contains supplementary material available at 10.1186/s12885-021-07787-8.

## Background

Transition is a critical period in the long-term follow-up (LTFU) care of childhood cancer survivors (CCSs). Transition generally describes the “purposeful, planned movement of adolescents and young adults with chronic physical and medical conditions from child-centered to adult-oriented health-care systems” [1]. This change in care is very important for CCSs, as the survival rate has increased substantially in recent decades [2], but survival is often associated with the risk of developing chronic medical and psychosocial late effects [3, 4]. Depending on the treatment received, these late effects can potentially affect any organ system [3, 5, 6]. The frequency of late effects increases with the time elapsed since the cancer diagnosis, and many late effects do not manifest until adulthood [3]. This carries the risk of adolescent and young adult CCSs no longer feeling the need for LTFU care [7, 8]. To prevent loss to follow-up and ensure the continuation of follow-up in adulthood, a well-organized and prepared transition process from pediatric- to adult-focused LTFU care is required. This transition can be part of and result in different LTFU care models for adult CCSs: a) models with transition to primary care physicians only, b) shared-care models with LTFU care provided by the primary care physician in collaboration with pediatric or adult oncologists, c) models with direct transition from pediatric to adult oncologists or d) models with transition to LTFU clinics, which provide LTFU care in multidisciplinary teams [9–11]. All these models have two aspects in common: they depend on physicians committed to LTFU care, and it can be challenging to find the right time to initiate the transition process and to perform the actual transfer. A proper assessment of CCSs’ individual transition readiness is needed. Survivor-related factors can influence transition readiness, such as CCSs’ personal responsibility concerning their health or knowledge of possible late effects and the reasons LTFU care is important [12]. To address these aspects, Klassen et al. developed and validated scales to measure CCSs’ transition readiness – the Cancer Worry scale (CWS) and the Self-Management Skills (SMSS) scale [13].

After officially translating both scales into German, we primarily aimed to assess the feasibility of using these scales in clinical practice and in the context of a different health care system than the one in which they were initially developed. Within this context, we aimed to assess the added value to transition readiness in Swiss CCSs aged ≥18 years.

## Methods

### Participants

Childhood cancer survivors ≥18 years old at participation; with ≥5 years since cancer diagnosis; who were enrolled in LTFU care at the Division of Oncology-Hematology, Department of Pediatrics, Kantonsspital Aarau; who planned to transition to adult care; and who had been younger than 18 years old at the time of their cancer diagnosis were eligible. We excluded CCSs who were not able to fill out the survey on their own due to language barriers or lacking the cognitive capacity.

### Assessment measures

The questionnaire used for this cross-sectional study consisted of two parts (Supplemental 1). The first part asked three demographic questions about cancer type, age at diagnosis, and treatment received. The second part corresponded to the translated CWS and the SMSS, originally developed by Klassen et al. [13]. The 6-item CWS focuses on the CCSs’ worries related to the cancer itself and to possible late effects. The 15-item SMSS assesses whether CCSs have enough skills to manage their own health. There are four possible answers to all questions, ranging from “strongly agree” to “strongly disagree”. The answers to the CWS can be transformed into a score ranging from 0 to 100, where higher scores show fewer cancer-related worries. No scoring system exists for the SMSS.

We obtained permission from McMaster University in Hamilton, Canada, to use the scales and translated them following a common translation procedure regulated by the Q-portfolio team (www.qportfolio.org). In the first step, two forward translators whose native language was German and who were fluent in English translated the scales from English to German independently of each other. The project manager (KS) combined these translations into a single version. A back translator whose native language was English and who was blinded to the original English version and was fluent in German then translated the combined version back into English. Five CCSs with different ages, diagnoses, sexes, and treatments tested the German version for understandability. Ethics approval to perform this study was obtained from the Ethikkommission Nordwest und Zentralschweiz (EKNZ).

### Recruitment

In January 2018, a combined transition clinic was established at Kantonsspital Aarau. This clinic is part of the LTFU care and is staffed by pediatric oncologists and adult oncologists and hematologists. If required, additional specialists in adult medicine participate in the consultation hours. The first visit to the transition clinic takes place in the children’s hospital. If the CCS feels ready, the following visit is located in the adult hospital but still attended by the pediatric team. During LTFU care, latest at the last visit before the transition, all CCSs receive an individualized survivorship care plan (SCP). The SCP summarizes the diagnosis, disease course and treatment received, including the cumulative doses of chemotherapeutic agents, radiotherapy, surgery, and information on hematopoietic stem cell transplantation. In addition, the SCP provides information about the individual risk of each CCS for late effects based on the treatment received and gives recommendations for follow-up examinations, including the interval. The goal of the transition clinic is to facilitate a slow and gradual transfer from pediatric to adult medicine. We integrated the questionnaire for this study into the transition clinic visit and asked the CCSs to participate after their annual LTFU consultation in the children’s hospital. After a verbal explanation of the study, a medical student handed out the study information and the informed consent form. After receiving the signed informed consent form, the questionnaire was filled out. The questionnaire was anonymous, and we did not gather further information from the CCSs’ medical records as this was a feasibility study only. We chose a random sample of 50 CCSs during a 9-month period.

### Analysis

We used descriptive statistics to describe the study population and summarize the answers to the CWS and SMSS. We transformed the answers of the CWS into the respective score ranging from 0 to 100 as mentioned in Klassen et al. [13]. We used STATA software (Version 16.0, Stata Corporation, Austin, TX) for the analysis.

## Results

### Characteristics of the study population

Most of the 50 CCSs participating in this study had been diagnosed with leukemia (46%), followed by lymphoma and solid tumors (18% each) (Table 1). The median age at cancer diagnosis was 10 years (interquartile range [IQR] 4–13), with nearly half of the CCSs diagnosed between 11 and 15 years of age. Most CCSs reported being treated with chemotherapy only (40%) followed by a combination of chemotherapy and surgery (24%) or additional radiotherapy (14%). Most CCSs remembered their cancer diagnosis (94%), and only 8% were not able to recall their age at diagnosis. All CCSs approached for this study confirmed their willingness to participate and filled out the questionnaire, and the participation rate was 100%. The questionnaire took the CCSs approximately 10 min to complete.

**Table 1:** Clinical characteristics of all participating childhood cancer survivors (*N* = 50)

	Participants n (%)
Cancer type	
Leukemia	23 (46)
Lymphoma	9 (18)
CNS tumors	6 (12)
Solid tumors	9 (18)
NA	3 (6)
Age at diagnosis	
Median (IQR) [years]	10 (4–13)
0–5	15 (30)
6–10	9 (18)
11–15	22 (44)
NA	4 (8)
Treatment modality	
Chemotherapy only	20 (40)
Chemotherapy and radiotherapy	6 (12)
Chemotherapy and surgery	12 (24)
Radiotherapy and surgery	2 (4)
Chemotherapy, radiotherapy and surgery	7 (14)
Chemotherapy, radiotherapy, surgery and transplantation	3 (6)

### Cancer worry scale (CWS)

All 50 CCSs answered all six items on the CWS and contradicted most of the statements with “Strongly disagree” or “Disagree”, which indicates a low to moderate level of cancer-related worry (Table 2). The median CWS score was 62 (IQR 55–71). Nevertheless, approximately 40% agreed or strongly agreed that they worry about possible late effects (44%), that they worry about not being able to have children in the future (40%), and that cancer is always in the back of their mind (42%). One-fourth of the CCSs worried about relapse (25%) and the development of a second malignancy (24%).

**Table 2:** Answers of the childhood cancer survivors to the Cancer Worry Scale; *N* = 50

	Strongly disagree n (%)	Disagree n (%)	Agree n (%)	Strongly Agree n (%)
1. I worry it might be difficult to have children in the future.	13 (26)	17 (34)	16 (32)	4 (8)
2. I worry about late effects that might happen to me (Note: late effects are health problems caused by cancer treatments, e.g. heart problems, hearing loss, learning problems).	6 (12)	22 (44)	16 (32)	6 (12)
3. Cancer is always at the back of my mind.	8 (16)	21 (42)	17 (34)	4 (8)
4. I worry about getting a new type of cancer.	13 (26)	25 (50)	10 (20)	2 (4)
5. I worry my cancer will come back (i.e. relapse).	16 (32)	21 (42)	11 (22)	2 (4)
6. I worry about my cancer every day	34 (68)	15 (30)	1 (2)	0 (0)

Score: Mean 61.9 (SD ±15.6)

Median 62 (IQR 55–71)

### Self-management skills scale (SMSS)

The answers to the SMSS indicate a relatively high level of autonomy of the survivors, with good self-management skills, as 63 to 100% of the CCSs’ answers were either “Agree” or “Strongly agree” (Table 3). Particularly in the areas of decision-making, communication, access to medical care and insurance, the participants show a high level of self-management. There were deficits and room for improvement in five areas in which less than 80% answered the items positively (Q5, Q9, Q11, and Q13). Three of these items assessed the CCSs’ level of responsibility and independence: one-third (36%) preferred to have the doctor speak to their parents instead of to them (Q9), one-fourth (24%) preferred to see a doctor or nurse together with their parents (Q11), and one-fifth (20%) did not book their appointments themselves (Q13). The fourth item (Q5) shows that nearly one-third (28%) of the participants disagreed or strongly disagreed with the statement: “I talk to a doctor or nurse when I have health concerns”. Furthermore, 16% denied talking to other people about their medical conditions (Q6). Five items were not answered by one or two participants (Q7, Q9, Q11, Q12, and Q15).

**Table 3:** Answers of the childhood cancer survivors to the Self-Management Skills Scale; *N* = 50

		Strongly disagree n (%)	Disagree n (%)	Agree n (%)	Strongly Agree n (%)	Missing n (%)
**Q1**	I answer a doctor or nurse’s questions.	0 (0)	0 (0)	17 (34)	33 (66)	0 (0)
**Q2**	I participate in making decisions about my health.	0 (0)	0 (0)	22 (44)	28 (56)	0 (0)
**Q3**	I make sure I go to all my doctor’s appointments.	0 (0)	1 (2)	12 (24)	37 (74)	0 (0)
**Q4**	I ask the doctor or nurse questions.	0 (0)	8 (16)	30 (60)	12 (24)	0 (0)
**Q5**	I talk to a doctor or nurse when I have health concerns.	2 (4)	12 (24)	23 (46)	13 (26)	0 (0)
**Q6**	I talk about my medical condition to people when I need to.	1 (2)	7 (14)	27 (54)	15 (30)	0 (0)
**Q7**	I am in charge of taking any medicine that I need.	0 (0)	3 (6)	18 (37)	28 (57)	1 (2)
**Q8**	I know how to contact a doctor if I need to.	0 (0)	1 (2)	21 (42)	28 (56)	0 (0)
**Q9**	I prefer it when a doctor speaks to me instead of my parent(s).	1 (2)	17 (34)	18 (36)	13 (26)	1 (2)
**Q10**	I can briefly describe my medical history when asked.	1 (2)	4 (8)	25 (50)	20 (40)	0 (0)
**Q11**	I prefer to see a doctor or nurse without my parent(s) with me.	1 (2)	11 (22)	19 (38)	18 (36)	1 (2)
**Q12**	I know how to access medical care when I travel.	0 (0)	2 (4)	29 (58)	18 (36)	1 (2)
**Q13**	I book my own doctor’s appointments.	1 (2)	9 (18)	20 (40)	20 (40)	0 (0)
**Q14**	I know the type of medical insurance I have.	0 (0)	7 (14)	25 (50)	18 (36)	0 (0)
**Q15**	I fill my own prescriptions when I need medicine.	1 (2)	6 (12)	20 (40)	21 (42)	2 (4)

## Discussion

Our study shows that the CWS and SMSS can be easily integrated into routine clinical visits. CCSs were very willing to complete the scales, and the time needed to complete them was short. The scales help clinicians to assess where each CCS individually stands in the transition process, in which areas additional support is needed, and in which areas the existing resources need to be strengthened.

### CWS in Swiss and Canadian CCSs and in the context of the existing literature

In a Canadian cross-sectional study, including 73 CCSs, the same scales were used [14]. Regarding type of cancer diagnosis, only data from 56 CCSs were available in the Canadian study. The distribution is largely comparable with our cohort for leukemia (46% vs 47%), but differs for lymphoma (18% vs 32%) and solid tumors. CNS tumors were not grouped separately in the Canadian cohort, but represented 12% of our cohort. Swiss CCSs showed lower levels of cancer-related worries than Canadian CCSs (mean CWS score in Swiss CCSs 61.9 ± 15.6; in Canadian CCSs 50.6 ± 18.4) [14]. For the vast majority of CCSs, cancer was not a worry that was present every day. Nevertheless, one in five CCSs worried about relapse or the development of a second tumor, and approximately 40% worried about possible late effects, including fertility issues. The worries about secondary tumors and late effects could potentially be increased by physicians’ increasing efforts to educate CCSs about reasons for attending and adhering to LTFU care. The explanation and handing over of the individualized survivorship care plan, which also includes surveillance recommendations for potential late effects, may cause worries. This shows that educating CCSs about possible late effects is a balancing act between “too much” education, which might lead to worries, and “too little” education, which probably creates fewer or no worries but results in a lack of knowledge of LTFU care. Informing patients is important, as a lack of knowledge can cause patients to become lost to follow-up [7, 15]. This underlines that worries need to be actively addressed. When handing over a document to the CCSs, such as an SCP, it is important to provide the contact details of someone who can be reached in case of questions.

### Results of the SMSS in the context of the existing literature

Participants showed an overall high level of self-management, as 80% or more agreed or strongly agreed with most of the statements (*n* = 11/15) on the SMSS. Three of the four statements (Q5, Q9, Q11, Q13) that indicated lower levels of self-management were linked to parental involvement (Q9, Q11, Q13) and reflected a certain dependency of the CCSs on their parents. This is congruent with other studies, which showed that some CCSs prefer parental involvement in the transition process and perceive this involvement as a facilitating factor [16, 17]. Parents of CCSs are used to taking on responsibility in the course of their child’s disease [12]. Therefore, CCSs first have to learn to manage their health care needs alone or with the parents in a supporting role but not taking the lead. In addition, parents sometimes struggle to hand over responsibility to their child [18], and CCSs admit receiving a certain benefit from ongoing parental support, such as making appointments, making decisions, and keeping important information [19]. One-quarter (24%) of CCSs preferred to see a doctor or nurse together with their parents. Comparing the answers to the questions about “speaking to the doctor” (Q9) and “seeing a doctor alone” (Q11), we saw that they were not congruent. Only seven CCSs (16%; 7/48) preferred to see a doctor or nurse together with their parents and also preferred when the doctor spoke to the parents instead of the CCS. This may indicate that some parents accompany the children for support and play a rather passive role during the clinical visit. This is supported by the results of the qualitative study (focus group) of Frederick et al., which showed that despite the participants’ desire to be perceived as an independent person, they mentioned the parental support, especially in administrative and organizational matters, as helpful [19]. Nevertheless, an early and active involvement of CCSs in their health management could help to prevent a too strong dependence on the parents and should start years before the transition [19–21]. In addition to promote independence in CCSs, it is equally important to support parents when they are transferring responsibility to their children. This leads to a gradual change from a patient-parent-physician triad to a patient-physician dyad. To achieve this change in role successfully, good communication is essential [19]. Our cohort was found to have room for improvement regarding communication. Only 72% of participants would talk to a doctor or nurse in case of health care concerns. Feeling comfortable asking questions and talking about health concerns is essential to become independent and improve self-management. One out of five CCSs (20%) reported that they did not book appointments themselves. Interestingly, seven CCSs who went to the appointments alone did not book the appointment themselves. This may indicate that these CCSs take responsibility for and have self-management skills regarding clinical visits, but administrative and organizational tasks are still taken care of by the parents. This result again goes in line with the findings of Fredericks et al., mentioned previously [19]. Patients at our hospital receive their planned appointment times in the mail several months in advance. Therefore, it is also possible that these patients reported that they did not make the appointment themselves because they received it from the hospital. The question about whether the CSSs booked appointments themselves (Q13) might not be valid in our population.

### SMSS in Swiss and Canadian CSSs

Regarding self-management, Swiss CCSs were less likely to address their health concerns to doctors, nurses, and other people than were Canadian CCSs and preferred when doctors spoke to their parents instead of them [14]. However, the proportion of Canadian CCSs who preferred to see the doctor or nurse together with their parents was slightly higher than that in the Swiss cohort (24% Swiss CCSs, 30% Canadian CCSs) [14]. Concerning health insurance and access to medical care, Swiss CCSs showed a relatively higher level of self-management than Canadian CCSs, as greater proportions of Swiss CCSs knew what type of medical insurance they have (86% vs. 63%) and how to access medical care when traveling (96% vs. 65%) [14]. Differences in health care systems and insurance between Switzerland and Canada might partially explain this situation. In Switzerland, children and adolescents up to the age of 18 years are insured through their parents; afterwards, it is their own responsibility, and they have to decide among multiple insurance options and providers. Health insurance always involves a so called “franchise” – a fixed deductible ranging from CHF 300 to 2′500. The franchise is the amount of a physician or hospital bill that must be paid in full by the insured party before the bills are partially paid by health insurance (max. 90% of the costs). However, the lower the franchise is, the higher the annual health insurance contribution. In Switzerland, health insurance is not paid by the employer nor is it deducted from an individual’s salary. In addition to health insurance, Switzerland has disability insurance. In cases of many specific types of childhood cancer, this insurance covers all costs, without a franchise, until the age of 20 years. In Canada, the universal health care system is publicly funded through taxes with separate health insurance plans for each province and territory [22, 23]. It is free for all Canadian citizens and gives access to basic medical services. Private insurance is needed for special services, such as drug prescriptions, dental care, home care or long-term care, physiotherapy, ambulance services or the prescription of glasses. The main difference between the Swiss and Canadian health care systems is that Swiss CCSs have to actively deal with health insurance, either when they are 18 years old or when they are 20 years old, and in Canada, everyone automatically has basic insurance. This may explain why Swiss CCSs’ knowledge of insurance was superior to that of Canadian CCSs.

### Strengths and limitations

The random sample of CCSs included in this study is broadly representative of 5-year survivors. Compared to the Swiss Childhood Cancer Survivor Study (SCCSS), which also included 5-year CCSs, our cohort had more patients with leukemia (46% vs 36%) but similar proportions of children diagnosed with lymphoma (18% vs 19%) and tumors of the CNS (12% vs 12%) [24]. We had a response rate of 100% and a very low number of unanswered questions. We think that social desirability bias played a minor role, since the evaluation also revealed critical responses and recruitment was done by a medical student who was not part of the treatment team.

The answers to the CWS might be biased because the CCSs completed the questionnaire directly after the visit. During the visit, already existing late effects were addressed and their possible occurrence in the future was mentioned. This could have activated worries that would otherwise not be present in the CCSs’ everyday lives.

## Conclusion

The translated CWS and SMSS are helpful tools in the assessment of transition readiness in Swiss CSSs, as they are easy to understand, quick to complete, and can be integrated into regular LTFU care.

Nevertheless, these questionnaires are not suitable as standalone instruments to determine a patient’s transition readiness. There would be a benefit to the longitudinal completion of these tools by CCSs to assess their advances in transition readiness and to determine the ideal individual time point for transition. Therefore, we will have all survivors, 15 years of age and older, fill out the scales at annual intervals in the future and will make it a regular component of LTFU care and the transition to adult-focused follow-up.

## Supplementary Information

**Additional file 1: Supplemental 1.** Questionnaire to assess transition readiness used in “Determining transition readiness in Swiss childhood cancer survivors – a feasibility study”.

## Data Availability

All data generated or analyzed during this study are included in this published article.

## Abbreviations

CCS: Childhood Cancer Survivor
CHF: Swiss Francs
CNS: Central nervous system
CWS: Cancer Worry Scale
IQR: Interquartile Range
LTFU: Long-term follow-up
SCP: Survivorship Care Plan
SMSS: Self-management Skill Scale
