# Supplementary material for: Determining transition readiness in Swiss childhood cancer survivors – a feasibility study
Source: BMC Cancer. 2021 Jan 21;21:84. doi: 10.1186/s12885-021-07787-8 (PMC7818544; doi:10.1186/s12885-021-07787-8)
Supplement: Supplementary file 1 — Additional file 1: Supplemental 1. Questionnaire to assess transition readiness used in “Determining transition readiness in Swiss childhood cancer survivors – a feasibility study”. [file 12885_2021_7787_MOESM1_ESM.docx]

**Supplemental 1: Questionnaire to assess transition readiness used in “Determining transition readiness in Swiss childhood cancer survivors – a feasibility study”**

Childhood cancer patients who have received treatment and follow-up care in a pediatric hospital often need long-term follow-up care and treatment as adults. Please refer to the meetings with your follow-up team for all statements below. Please also fill out the first section on basic information about your medical history.

Type of cancer diagnosis: ☐ Leukemia ☐ Tumors of the CNS ☐ Lymphoma

☐ Neuroblastoma ☐Nephroblastoma ☐Germ cell tumor

☐ Others: ____________________________________

Age at cancer diagnosis: Treatment received: ☐ Chemotherapy

- Radioterhapy
- Surgery
- Transplantation

**Cancer Worry Scale**

These questions are about **thoughts and feelings you may have as a cancer survivor**. For each question, please circle **only 1 answer**.

|  | **Strongly disagree** | **Disagree** | **Agree** | **Strongly agree** |
| --- | --- | --- | --- | --- |
| I worry it might be difficult to have children in the future. | ☐ | ☐ | ☐ | ☐ |
| I worry about late effects that might happen to me (Note: late effects are health problems caused by cancer treatments, e.g. heart problems, hearing loss, learning problems). | ☐ | ☐ | ☐ | ☐ |
| Cancer is always at the back of my mind. | ☐ | ☐ | ☐ | ☐ |
| I worry about getting a new type of cancer. | ☐ | ☐ | ☐ | ☐ |
| I worry my cancer will come back (i.e. relapse). | ☐ | ☐ | ☐ | ☐ |
| I worry about my cancer every day | ☐ | ☐ | ☐ | ☐ |

**Self-Managemen Skill Scale**

These questions are about being **in charge of your health**. For each question, please circle **only 1 answer**.

|  | **Strongly disagree** | **Disagree** | **Agree** | **Strongly Agree** | **Missing** |
| --- | --- | --- | --- | --- | --- |
| I answer a doctor or nurse’s questions. | ☐ | ☐ | ☐ | ☐ | ☐ |
| I participate in making decisions about my health. | ☐ | ☐ | ☐ | ☐ | ☐ |
| I make sure I go to all my doctor’s appointments. | ☐ | ☐ | ☐ | ☐ | ☐ |
| I ask the doctor or nurse questions. | ☐ | ☐ | ☐ | ☐ | ☐ |
| I talk to a doctor or nurse when I have health concerns. | ☐ | ☐ | ☐ | ☐ | ☐ |
| I talk about my medical condition to people when I need to. | ☐ | ☐ | ☐ | ☐ | ☐ |
| I am in charge of taking any medicine that I need. | ☐ | ☐ | ☐ | ☐ | ☐ |
| I know how to contact a doctor if I need to. | ☐ | ☐ | ☐ | ☐ | ☐ |
| I prefer it when a doctor speaks to me instead of my parent(s). | ☐ | ☐ | ☐ | ☐ | ☐ |
| I can briefly describe my medical history when asked. | ☐ | ☐ | ☐ | ☐ | ☐ |
| I prefer to see a doctor or nurse without my parent(s) with me. | ☐ | ☐ | ☐ | ☐ | ☐ |
| I know how to access medical care when I travel. | ☐ | ☐ | ☐ | ☐ | ☐ |
| I book my own doctor’s appointments. | ☐ | ☐ | ☐ | ☐ | ☐ |
| I know the type of medical insurance I have. | ☐ | ☐ | ☐ | ☐ | ☐ |
| I fill my own prescriptions when I need medicine. | ☐ | ☐ | ☐ | ☐ | ☐ |
